# Arecolin in Colic1Read at the January meeting of the Keystone Veterinary Medical Association.

**Published:** 1898-03

**Authors:** S. J. J. Harger

**Affiliations:** Veterinary Department, University of Pennsylvania


### ARECOLIN IN COLIC.[1]

By S. J. J. Harger, V.M.D.,
VETERINARY DEPARTMENT, UNIVERSITY OF PENNSYLVANIA.

Among the drugs employed to produce intestinal peristalsis for relief from abdominal pain due to intestinal obstruction, tympanitis, constipation, or the presence of irritating intestinal contents, certain preparations are well known. The first agents that promised to be almost specifics, either separately or in combination, were eserin and pilocarpin, and more recently barium chloride. None of these are specifics, and sometimes fail to act when their action is most desired. Another preparation for which marked peristaltic excitant properties are claimed is arecolin.

Arecolin is an alkaloid extracted from the seed of the *areca catechu,* or betel-nut. The seed is light brown and cone-shaped, and the plant is found in equatorial Asia and the adjoining islands. Jahns more recently made a chemical study of the powdered seed, and has isolated a tannin and three alkaloids: 1, arecolin; 2, arecaine; 3, an alkaloid of minimum quantity, whose existence is denied by some chemists (Mouquet). Arecaine is supposed to be toxic.

The only one of these three alkaloids that is of any interest to veterinary medicine is arecolin, which in its physic, chemic, and pharmaceutic properties resembles the alkaloid derived from pomegranate root.

It is soluble in water, alcohol, and chloroform, and with acids forms very soluble salts. Of the latter, the hydrobromate and hydrochlorate are the only ones employed. The hydrobromate is deliquescent, and is, therefore, less desirable for therapeutic use

[1] Read at the January meeting of the Keystone Veterinary Medical Association.

than the hydrochlorate, and is said not to produce the effects of arecolin as effectively as the hydrochlorate salt does.

The therapeutic value and the superior merits of this agent over others used for the same purposes are not absolutely determined, and can only be established by its employment in practice. Some experiments, however, are recorded.

*Physiologic Action.* Injected subcutaneously, the hydrobromate of arecolin has no local action. Its systemic action is similar to that of eserin and pilocarpin combined; the effect upon the secretions is marked. There is an intense salivation in from four to six minutes after the injection, and continues for about thirty minutes.

Large doses of arecolin produce physiologic effects similar to those of muscarine: arrest of respiration and muscular contractility, and more especially in the dog and the cat is this action decided.

Fifty to seventy milligrammes (one grain) of either of the two salts injected into a dog of four to five kilos (eight to ten pounds) provoke irritability of the heart and tetanic spasms, followed by partial paralysis, but not always by death, unless these symptoms are accompanied by vomiting and liquid alvine discharges.

In cold-blooded animals arecolin in large doses produces excitation of reflex action, convulsions, and then paralysis. In the frog it arrests the heart in diastole. In these animals, also, according to the same authors (Battistini and Scofone), it excites myosis, salivation, diarrhœa, and finally cardiac and pulmonary paralysis.

In small doses arecolin diminishes the heart-beats, influences the rhythm, and increases the number of respirations, excites intestinal peristalsis in the same manner as eserin does, and causes a marked hypersecretion of all the glands in the digestive tract. It is, therefore, an intestinal evacuator more certain than eserin. The intensity of this action is dependent upon the quantity of the alkaloid employed, and is but little marked if the dose given is below a certain mean, about two centigrammes (one-third of a grain hypodermically) for a horse of average weight (Gobbels).

The symptoms produced by the salts are analogous to the above: cardiac irritability, muscular trembling, tetanic spasms, muscular paralysis, difficult respiration, dyspnœa, syncope, asphyxia, cardiac paralysis, and contraction of the pupil; pin-point pupil in the dog. The antidote for arecolin poisoning is atropia.

Fröhner has made a special study of this alkaloid, and, according to him, it is an active sialagogue in one-tenth to one-half gramme doses (one and one-half to seven grains).

Its laxative action is equivalent to eserin and pilocarpin; it is about ten times as active as the latter and about equal to the former. In doses indicated above it acts in fifteen to thirty minutes after the injection; its purgative action is accompanied by colicky pain, and in some instances nausea; large doses produce perspiration and abundant emission of urine. Twenty-five grammes in the horse produced epileptiform convulsions, and fifty grammes killed the animal, death being preceded by tetanic spasms, as seen in nicotin and strychnine poisoning. Arecolin is, therefore, a very active agent whose effects are not without danger if the dose employed be excessive.

Arecolin is very rapidly eliminated from the body with all the secretions and excretions, and successive (one hour) doses are, therefore, easily borne by the animal.

*Therapeutic Uses.* Maume, in 1889, called attention to the action of arecolin upon the secretions, the intestinal peristalsis, and the muscular system in general. Most authors agree that it should have the preference in cases in which eserin and pilocarpin combined are indicated. It is fully as active as eserin, and possesses eight to ten times the strength of pilocarpin; it is more stable than eserin, is more readily preserved, and is more moderate in price. Fröhner has employed it extensively as an excitant for intestinal peristalsis when a rapid evacuation of the intestines was desired, as in colic, obstruction, constipation, etc.; also in the horse when obstruction of the œsophagus, as from carrots, beets, etc., and in verminous colic. Mouquet has experienced the same advantages with arecolin in cases of intestinal indigestion in the horse. And this method of treating non-inflammatory conditions of the intestines accompanied by colicky pain appears to be the most rapid as well as the most rational, and the drug which proves itself to accomplish this object in the shortest time, and with the least danger to the life of the animal, should have the preference.

Arecolin has been used in acute laminitis, acting as a revulsive in drawing the blood from the extremities toward the centre of the body, and favoring the absorption of the material exuded over the podophyllous tissue. Paiman confirms the preceding indication. A horse suffering from laminitis for twenty-four hours was radically cured after four injections of one gramme of hydrobromate of arecolin at intervals of an hour. In a second animal the disease had existed for four days, and the cure was completed after three injections. Arecolin is contraindicated in horses with respiratory troubles or with functional alterations of the heart; also in pregnant females. Common horses appear to be more susceptible to the action of this alkaloid than well-bred horses. Cattle, it appears, are also very susceptible.

For the horse the dose is one-half to three-fourths of a grain hypodermically. Rather than give a larger quantity, the dose may be repeated. The dog is less sensitive to the alkaloid than the horse. The dose is one-sixth of a grain for a large dog, and proportionately less for a small one.

Many drugs have been placed upon the market and given promise of good results, but our labors were only rewarded by disappointment. I will, therefore, not be too sanguine in my statements, and must admit that further experimentation is necessary to realize the promised advantages of this agent. It is well worth the attempt to prove or disprove the merits which this preparation is supposed to possess.

